# SERS-Based Aptasensor for Rapid Quantitative Detection of SARS-CoV-2

**DOI:** 10.3390/nano11061394

**Published:** 2021-05-25

**Authors:** Elena Zavyalova, Oganes Ambartsumyan, Gleb Zhdanov, Dmitry Gribanyov, Vladimir Gushchin, Artem Tkachuk, Elena Rudakova, Maria Nikiforova, Nadezhda Kuznetsova, Liubov Popova, Bakhtiyar Verdiev, Artem Alatyrev, Elena Burtseva, Anna Ignatieva, Anna Iliukhina, Inna Dolzhikova, Alexander Arutyunyan, Alexandra Gambaryan, Vladimir Kukushkin

**Affiliations:** 1 Chemistry Department, Lomonosov Moscow State University, 119991 Moscow, Russia; gleb.zhdanov@chemistry.msu.ru; 2 Department of Microbiology, Virology and Immunology, I.M. Sechenov First Moscow State Medical University, 125009 Moscow, Russia; ogan-mail@mail.ru; 3 Institute of Solid State Physics of Russian Academy of Science, 142432 Chernogolovka, Russia; digrib@gmail.com; 4 National Research Center for Epidemiology and Microbiology Named after the Honorary Academician N. F. Gamaleya, 123098 Moscow, Russia; wowaniada@gmail.com (V.G.); artem.p.tkachuk@gmail.com (A.T.); marianikiforova@inbox.ru (M.N.); nadyakuznetsova0@gmail.com (N.K.); ljubovprokudina@gmail.com (L.P.); yuryevpolskei@yandex.ru (B.V.); artem.alatyrev@gmail.com (A.A.); elena-burtseva@yandex.ru (E.B.); valgella@yandex.ru (A.I.); sovanya97@yandex.ru (A.I.); i.dolzhikova@gmail.com (I.D.); 5 Institute of Physiologically Active Compounds of Russian Academy of Science, 142432 Chernogolovka, Russia; evladru@mail.ru; 6 Belozersky Research Institute of Physico-Chemical Biology, Lomonosov Moscow State University, 119991 Moscow, Russia; Alarut@genebee.msu.ru; 7 Chumakov Federal Scientific Center for Research and Development of Immune and Biological Products RAS, 108819 Moscow, Russia; al.gambaryan@gmail.com

**Keywords:** SARS-CoV-2, COVID-19, SERS, silver colloids, aptamer, optical sensor, respiratory viral infections

## Abstract

During the COVID-19 pandemic, the development of sensitive and rapid techniques for detection of viruses have become vital. Surface-enhanced Raman scattering (SERS) is an appropriate tool for new techniques due to its high sensitivity. SERS materials modified with short-structured oligonucleotides (DNA aptamers) provide specificity for SERS biosensors. Existing SERS-based aptasensors for rapid virus detection are either inapplicable for quantitative determination or have sophisticated and expensive construction and implementation. In this paper, we provide a SERS-aptasensor based on colloidal solutions which combines rapidity and specificity in quantitative determination of SARS-CoV-2 virus, discriminating it from the other respiratory viruses.

## 1. Introduction

In late 2019 in Wuhan (Hubei, China), a new strain of coronavirus SARS-CoV-2 was first detected, which caused a pandemic of acute respiratory disease, named coronavirus disease 2019 (COVID-19). The virus is rapidly spread and has already claimed the lives of more than two million people all over the world. It also made a significant impact on global economy, put pressure on healthcare, and affected social life. The disease is characterized by progressive inflammatory pulmonary cellular infiltration, hypoxemia, and the development of an acute respiratory distress syndrome (ARDS), which could lead to death.

The situation is complicated by the fact that many infected patients have very mild symptoms or are completely asymptomatic during the entire period of infection, but can still transmit the disease to others [1,2]. Additionally, symptoms of COVID-19 are similar to other respiratory illnesses, so it needs to be differentiated from bacterial and other viral pneumonia caused by influenza virus, adenovirus, parainfluenza, or respiratory syncytial virus (RSV) for clinical diagnostic [3].

Traditional detection methods such as quantitative polymerase chain reaction with a reverse transcription (RT-qPCR) and enzyme-linked immunosorbent assay (ELISA) require qualified personnel, laboratory with separate areas, often expensive reagents, and complicated sample preparation (especially for the PCR), in which errors can lead to false positive or false negative results [4,5]. Lateral flow assay (LFA) tests are also widespread due to the COVID-19 pandemic. Their advantages include speed of analysis, as well as no need for qualified personnel and a special room; therefore, a person can test himself for disease. However, these tests can give false negative results more often as compared to the above methods due to their lower sensitivity [6,7,8].

Due to rapid spread of COVID-19 around the world and similar symptoms to other viral and bacterial pneumonia, an interest in the development of fast, easy-to-use, and selective methods for biosensor diagnostics of respiratory diseases has increased.

Biosensors present a great interest for scientists and clinicians in diagnostics due to their advantages over the existing laboratory methods such as RT-qPCR and antibody-based techniques (enzymatic, fluorescent, and chemiluminescent). They have similar sensitivity but require less time and no advanced laboratory equipment with qualified personnel. They are also as fast but more sensitive than LFAs [9].

Among the promising devices, there are biosensors based on surface-enhanced Raman scattering (SERS). They can be classified into label-free and reporter-based platforms. The using of label-free schemes is limited because, usually, biological object is analyzed in a mixture of other biological substances, which causes overlapping of spectral lines and difficulty in identifying substances [10]. More promising is using commercially available Raman-active dyes, which are widely used as reporter molecules [11,12]. Another important component of the biosensor is recognizing elements such as antibodies or aptamers, which allows capture of certain objects from a biological mixture.

This capture, in turn, results in a change of a certain physical property, not limited to a change in Raman spectra [13]. Moreover, the introduction of biosensors on the surface of plasmonic nanoparticles often leads to optical detectors that, upon binding, make the target change itscolour [14]. In recent years, there has been a growing interest in using aptamers as biorecognizing elements. In particular, aptamer-based biosensors provide a great level of specificity and variability of modifications. Aptamers are an alternative to antibodies for their comparable qualities in detection [15].

For the existing SERS-based aptasensors for detection of respiratory viruses, there are several published approaches. Negrie et al. suggested an application of a polyvalent DNA aptamer to influenza nucleoprotein. Sigmoidal dependence of direct SERS intensity was a function of aptamer concentration on Ag substrate from 1 to 5000 nM. The binding of the target and aptamer changed a secondary structure, which was sensed by SERS. Incubation with a viral content took more than 8 h for registering SERS spectra of the aptamer, which limits practical implementation [16]. In the latest works devoted to influenza detection, Chen et al. developed an impressive aptasensor for influenza A (H1N1) with LOD of 97 PFU/mL and 20 min per an assay with a reverse monotonous dependence on a viral load. However, preparation of an advanced 3D nano-popcorn SERS substrate demanded more than 7 h [17].

For the newest aptasensors for SARS-CoV-2, Stanborough et al. compared SPR, BLI, and SERS in performance of direct spike protein detection. SERS-detection showed the lowest LOD of 1 fM and dynamic range of six orders. It required more than 2 h of sample preparation and did not imply whole viral particles detection, which has less clinical significance [18]. Yang et al. [19] presented a human angiotensin-converting-enzyme-2functionalized gold “virus traps” nanostructure as a very sensitive SERS biosensor. This optical sensor was used for selective capture and rapid detection of S-protein (expressed SARS-CoV-2) in the contaminated water.

In our previous work, we compared the use of solid-state SERS substrates and colloidal SERS particles to detect influenza A virus. On solid-state SERS substrates, we managed to achieve LOD for influenza A virus of 10^4^ VP/mL with less than 15 min for an assay. We applied SERS substrate with immobilized primary DNA aptamer RHA0385 with broad specificity towards to influenza A virus hemagglutinin on silver and secondary labeled aptamer for “sandwich” assembly. This approach allowed to detect whole viral particles with hemagglutination activity. However, the dependence was not monotonous, which allowed only qualitative interpretation of the result [20].

In our second work of influenza detection, we switched from the solid SERS substrate to the colloidal one with the same “sandwich” approach and aptamers. This allowed us to create a simple and rapid “one-pot” technique with a monotonous dependence but with a modest dynamic range of 2 × 10^5^ − 2 × 10^6^ VP/mL [21].

For this work, we used a colloidal SERS-based aptasensor with a new setup including changes in nanoparticle aggregation, aptamer, and target virus, i.e., SARS-CoV-2 virus. The aptamer was RBD-1C, which was shown to have high affinity to receptor binding domain (RBD) of surface S-protein of SARS-CoV-2 [22].

## 2. Materials and Methods

### 2.1. Procedure of AgNPs Synthesis

Preparation of the silver colloids by reducing a silver nitrate solution with hydroxylamine hydrochloride was conducted in concordance with the method [23]. Silver nitrate (AgNO_3_, CAS 7761-88-8), hydroxylamine hydrochloride (NH_2_OH-HCl, CAS 5470-11-1) were of the highest purity available; sodium hydroxide (NaOH, CAS 1310-73-2), sodium chloride (NaCl, CAS 7647-14-5), xanthine (CAS 69-89-6)were analytical-grade and used without further purification. All the products were purchased from Aldrich (Sigma-Aldrich Inc., St. Louis, MO, USA). The stock solutions of reagents and silver sols were prepared with water from a Milli-Q system (18.2 MΩ·cm resistivity at 25 °C).

The AgNPs were prepared at room temperature under vigorous stirring by rapid addition of a small volume of a concentrated silver solution (10 mL) to a large volume of a less-concentrated hydroxylamine hydrochloride/sodium hydroxide solution (90 mL). The concentration of AgNO_3_ and NH_2_OH-HCl/NaOH was 10^−3^ M and 1.5 × 10^−3^/3 × 10^−3^ M, respectively, in the final reaction mixture. It was kept under stirring for 30 min once the addition of silver nitrate was complete. A yellow-brown colored transparent colloidal suspension with no sediment was obtained. The synthesized colloids were stable for several weeks under refrigeration conditions at +6 − +8 °C.

UV-Vis spectra of AgNPs were recorded on a spectrometer Genesis 10S UV-Vis (Thermo-Fisher Scientific, Madison, WI, USA). The morphology of silver nanoparticles was studied by SEM (Scanning Electron Microscopy), performed using a Supra 50VP electron microscope (Zeiss, Germany) with a resolution of 1.5 nm. The mean size and ζ-potential of AgNP were determined by DLS (Dynamic Light Scattering), performed using Zetasizer Nano ZS (Malvern, Worcestershire, UK).

### 2.2. Viruses

Vero E6 (ATCC CRL-1586) cell line was obtained from the Chumakov Federal Scientific Center for Research and Development of Immune and Biological Products RAS, Russia, and was maintained in complete Dulbecco’s modified Eagle’s medium (DMEM), containing 10% fetal bovine serum (FBS, HyClone, Logan, Utah, USA), L-glutamine (4 mM), and penicillin/streptomycin solution (100 IU/mL; 100 μg/mL) (PanEco, Moscow, Russia). SARS-CoV-2 isolate PMVL-5 (GISAID accession EPI_ISL_470899) was isolated in May 2020 from a nasopharyngeal swab specimen taken from a 22-year-old female. The nasopharyngeal swab was inoculated on Vero E6 (non-human primate kidney) cells. The inoculated cells were monitored for cytopathic effects by light microscopy and cytopathic effects were detected at 72 h post inoculation. Virus was passaged three times before the experiments to pile the virus. Viral titer of 4.6 × 10^6^ TCID50/mL was determined as TCID50 by endpoint dilution assay. Virus titer was calculated using the Reed and Muench method [24]. All experiments with the live SARS-CoV-2 followed the approved standard operating procedures of the NRCEM biosafety level 3 facility.

Influenza viruses and Newcastle disease virus were provided by the Chumakov Federal Scientific Center for Research and Development of Immune and Biological Products of the Russian Academy of Sciences. The following strains were studied: influenza A virus (IvA) A/FPV/Rostock/34 R6p (256 HAU/mL in stock solution); influenza B virus (IvB) B/Victoria/2/1987 (2000 HAU/mL in stock solution); Newcastle disease virus(NDV) (256 HAU/mL in stock solution). Virus stocks were propagated in the allantoic cavity of 10-day-old embryonated specificpathogen-free chicken eggs. Eggs were incubated at 37 °C, cooled at 4 °C 48 h post-infection, and harvested 16 h later. The study design was approved by the Ethics Committee ofthe Chumakov Institute of Poliomyelitis and Viral Encephalitides, Moscow, Russia (Approval #4 from 2 December 2014). Viruses were inactivated via the addition of 0.05% (*v*/*v*) glutaric aldehyde, preserved via the addition of 0.03% (*w*/*v*) NaN_3_, and stored at +4 °C.

Adenovirus type 6 (AdV) Strain Tonsil 99 (Bialexa, Russia) and respiratory syncytial virus (RSV) (Bialexa, Russia) were inactivated by treatment with Thimerosal and beta propiolactone. Viral content of RSV was 1 mg/mL (2·10^12^ VP/mL); viral content of AdV was 1.9 mg/mL (4·10^12^ VP/mL). Viral particle concentrations (VP/mL) were calculated from the protein concentration.

### 2.3. Aptamers and Their Assembly

The following oligonucleotides were studied. Biotin-RBD-1C (Biotin-5′-CAGCAC CGACCTTGTGCTTTGGGAGTGCTGGTCCAAGGGCGTTAATGGACA-3′ from Synthol, Moscow, Russia), Biotin-RBD-1C-sh (Biotin-5′-TTTGGGAGTGCTGGTCCAAGGG CGTTAATGGACA-3′ from Synthol, Moscow, Russia), BDP FL-RBD-1C (Bodipy FL-5′-CAGCACCGACCTTGTGCTTTGGGAGTGCTGGTCCAAGGGCGTTAATGGACA-3′ from Lumiprobe, Moscow, Russia). To assemble the structure of the aptamers, the following algorithm was used. Biotinylated aptamers were prepared in 2 µM concentrations in the buffer containing 10 mM tris-HCl pH 7.0, 140 mM NaCl, and 10 mMKCl. Bodipy FL labeled aptamer was prepared in 2 µM concentrations in the buffer containing 10 mM PBS. The solutions were heated at 95 °C for 5 min and cooled at room temperature.

### 2.4. Circular Dichroism and UV-Spectroscopy

A 2 μM aptamer solution was placed in a quartz cuvette with 1 cm path. Circular dichroism (CD) spectra were acquired using CD spectrometer Chirascan (Applied Photophysics, Leatherhead, UK) and a dichrograph MARK-5 (Jobin-Yvon; Montpellier, France) equipped with a thermoelectric temperature regulator. The spectra were acquired in the range of 220–360 nm.

### 2.5. Determination of Aptamer Affinity to S-Protein and Binding to SARS-CoV-2 Virus

Kinetic constants of association and dissociation of the RBD-S-protein complexes were determined using interferometer Blitz (Forte-Bio, Fremont, CA, USA). Streptavidin biosensors from Forte-Bio (USA) were used. The biosensors were hydrated for 10 min in the buffer. The biotinylated aptamer was loaded on the biosensor from 1 µM solution for 120 s. The binding experiments were performed as following:

(1) baseline in the buffer during 30 s;
(2) association stage in 80–1200nM RBD of S-protein (from HyTest, Turku, Finland) solution during 200 s;
(3) dissociation stage in the buffer during 300 s.

Kinetic constants were calculated from exponential approximations of the curves [25]. Several experiments were performed to test the affinity of the aptamers to inactivated SARS-CoV-2 virus, including experiments on Photonic Crystal Surface Mode (PC SM)-based biosensor “EVA 2.0” (Institute of Spectroscopy, Russian Academy of Sciences, Troitsk, Moscow, Russia).

The experiments on “EVA 2.0” with viruses were performed as following:multilayered glass SiO_2_|Ta_2_O_5_|SiO_2_ was silanized with APTES from the one side by hydrolisation. The other side of the glass was treated with oil to the panel of the device with a red laser. A glass cell with hosepipes was installed on top of the glass. A disc pump was connected to the outlet hose. At the beginning of the experiment, bidistilled water was run through the entire system to avoid the bubbles’ formation and to elevate of measurement accuracy. The ideal interference pattern obtained could be seen through the video camera set up on the device.

The silanized side of the glass was treated with the following solutions at room temperature:

(1) An amount of 0.1 M NaH_2_PO_4_ pH = 6.2 for 2 min.
(2) The activation of -NH_2_ groups of APTES by EDC (50 mg/mL) and NHS (50 mg/mL) for 20 min.
(3) An amount of 50 mM MES pH~5.0 for 5 min.
(4) A covalent conjugation of streptavidin to the activated -NH_2_ groups. Streptavidin (40 µg/mL) in 50 mM MES pH~5.0 for 30 min.
(5) Washing with 50 mM MES pH~5.0 for 2 min.
(6) Washing with 5 mM glycine in 25 mM HCl (pH~2) for 1–2 min.
(7) Washing with 10 mM Tris-HCl (pH = 7.4), 140 mM NaCl, 10 mM KCl for 5 min.
(8) Loading of biotinylated aptamer to SARS-CoV-2 (RBD-1C) for 15–20 min.
(9) Washing with 10 mM Tris-HCl (pH = 7.4), 140 mM NaCl, 10 mM KCl for 5 min.
(9a) Interaction with cell medium for 3 min.
(9b) Interaction with SARS-CoV-2 virus with a titer of 0.22·10^6^ TCID50/mL or 0.11·10^6^ TCID50/mL for 3 min.
(10) Dissociation in 10 mM Tris-HCl (pH = 7.4), 140 mM NaCl, 10 mM KCl for 5 min.

### 2.6. SERS Measurements

The SERS spectra were acquired using a handheld Raman analyzer RaPort (Enhanced Spectrometry, Inc., San Jose, CA, USA) with a laser wavelength of 532 nm and a working output power of 38 mW. The spectrometer had a spectral resolution of 4–6 cm^−1^ and a spectral range of 160–4000 cm^−1^. The recording time of a single spectrum was 400 ms with 20 averages. The measurements were carried out in glass vials with a volume of 1.5 mL (Akvilon, Moscow, Russia). The focus of the laser beam coincided with the center of the vial.

We took 150; 50; 25; 12.5; 9; 7.5; 6; 4.5; 3; 1.5; 0.75; 0.37; and 0.15 µl of a cell culture medium with SARS-CoV-2, control viruses (RSV, IvA, IvB, NDV, AdV), or a virus-free cell culture medium. The cell culture medium was the same for all viruses for a more objective comparison. The control viruses were prepared in a concentration of 10^8^ VP/mL, supposing that the ratio between TCID50/mL and VP/mL is about 1 to 100 as was estimated for fresh influenza viruses by Kramberg et al. [26]. Then, we added 4 µL of 2 µM BDP FL-RBD-1C. For the volumes of 25 µL and less, we added PBS to obtain a total volume of 50 µL before the addition of the labeled aptamer. After 5 min of the incubation, we added 250 µL of PBS and injected the solution to 196 µL of AgNPs (the total volume was 500 µL). SERS spectra was registered at 1 min after the aggregation step.

## 3. Results

### 3.1. Characterization of Silver Colloidal Nanoparticles

In this work, we used hydroxylamine-reduced AgNPs [23] as SERS substrate. These silver colloids are stable and highly SERS-active with good reproducibility in the obtained enhancement factors, as well as having the possibility of long-term (about a month) storage without loss of efficiency.

To characterize the morphology of the produced colloids, UV-Vis spectroscopy (Figure S1) and DLS (Figure S2) were used. The absorption maximum of the measured UV-Vis spectrum of the colloidal solution provides information on the average particle size, whereas its full width at half-maximum (FWHM) can be used to estimate particle dispersion. The absorption maximum was found at 394 nm with a FWHM of approximately 55 nm. The mean size according to the number distribution of DLS is 4.8 nm, whereas the intensity distribution gives the size of 44 nm, being in agreement with the value calculated from spectral characteristics of NP. These results have been confirmed by SEM (Figure S3). The AgNPs are supposed to have no interaction with the aptamer as both have strong negative charge. ζ-potential of NPs is −54 + −18 mV (Figure S4).

Xanthine, purine base, was selected as a test compound to investigate the effectiveness of silver colloids in enhancing SERS spectra. The spectra of the test substance xanthine at different ionic strengths of the solution are shown in Figure S5.

### 3.2. Structural Characterization of Aptamer RBD-1C

**Figure 1:**
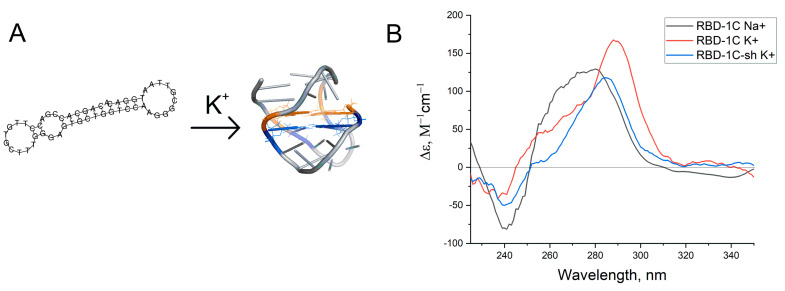
(**A**) Putative structures of the aptamer RBD-1C. (**B**) Circular dichroism spectra of RBD-1C in 140 mM NaCl (‘Na^+^’), RBD-1C, and RBD-1C-sh in 140 mM NaCl and 10 mM KCl (‘K^+^’).

The structure of the aptamer RBD-1C was supposed by Song et al. [22]; it consists of two hairpins that are end-to-end stacked (Figure 1A). The RNA fold webserver [27] provided the same folding for the sequence of RBD-1C. DNA duplexes are known to have hyperchromic effect during unfolding in UV-spectra at the wavelength of 260 nm [28]. However, no notable changes in UV-spectra were tracked during RBD-1C melting in potassium buffer (Figure S6). Circular dichroism (CD) spectra were recorded to identify the other possible topologies of RBD-1C (Figure 1B).

The spectra contained a positive peak at 290 nm, a shoulder at 254 nm, and a slight negative peak at 230 nm. These CD spectra could not be attributed to duplexes [29]; similar spectra were reported for two-quartet antiparallel G-quadruplex with a diagonal loop (Figure 1A) [30]. CD spectra in sodium buffer had a shoulder at 290 nm and major peak at 270–280 nm that correspond to a preferable DNA duplex. Thus, an equilibrium between two conformations was observed. A shortened version of the aptamer was studied to reveal whether G-quadruplex is a recognizing element in this aptamer. Seventeen nucleotides from the 5′-end of the aptamer were excluded (oligonucleotide RBD-1C-sh). This oligonucleotide had CD spectra that are close to RBD-1C. Both oligonucleotides were studied in affinity experiments.

### 3.3. Affinity of RBD-1C and Its Shortened Version to RBD of S-Protein and the Whole Virus

**Figure 2:**
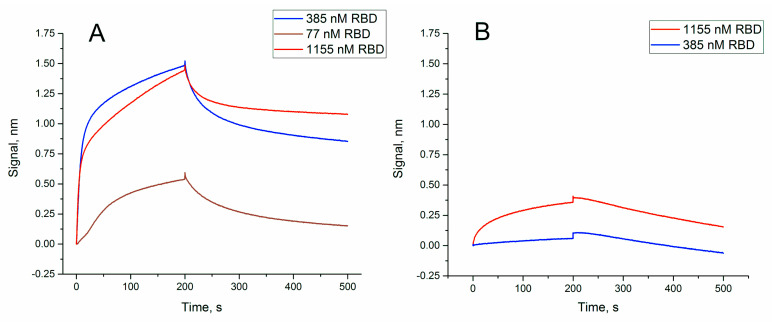
Sensorgrams of the interaction between soluble RBD of S-protein and immobilized oligonucleotides: (**A**) RBD-1C; (**B**) RBD-1C-sh. The concentrations of protein are provided in the graphs.

The affinity of RBD-1C to the recombinant protein has been previously estimated by Song et al. [22]. Here, we estimated it once more and compared the affinities of RBD-1C with its truncated variant. The sensorgrams are shown in Figure 2.

Dissociation constant of RBD-1C was 13 ± 2 nM (Table 1),which agrees well with the previous value of 5.8 ± 0.8 nM [19]. The truncated variant, RBD-1C-sh, had a dissociation constant of 330 ± 60 nM that corresponds to low specific binding.

**Figure 3:**
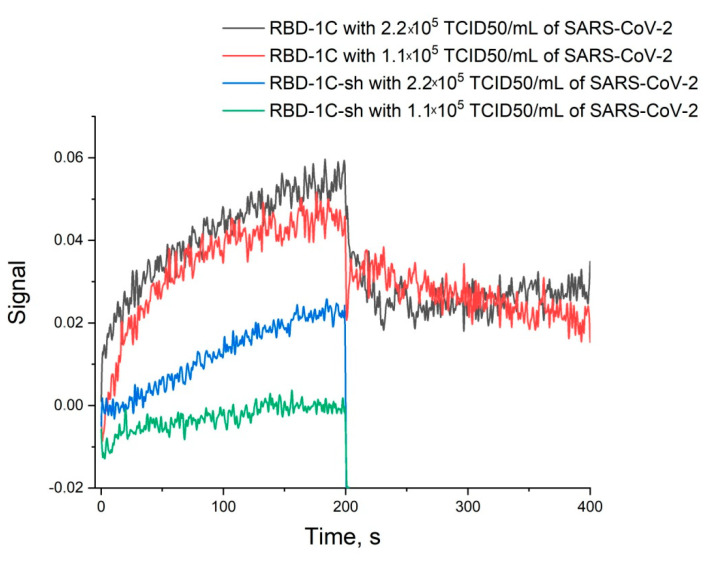
Sensorgrams of the interaction between SARS-CoV-2 viruses and immobilized oligonucleotides RBD-1C and RBD-1C-sh. The concentrations of the virus are provided.

Thus, the unique spatial structure of RBD-1C is crucial for high affine recognition of the RBD of S-protein. Next, the ability of binding the whole virus was tested for both RBD-1C and RBD-1C-sh. RBD-1C showed excellent curves for both concentrations of the virus; whereas RBD-1C-sh provided low-efficient binding of the viruses (Figure 3). It could be concluded that the active aptamer structure is a DNA hairpin of RBD-1C, whereas G-quadruplexes are low-active additives. The content of the virus was estimated in TCID50 that corresponds to virulent particles.

### 3.4. Specificity of SERS Aptasensor

**Figure 4:**
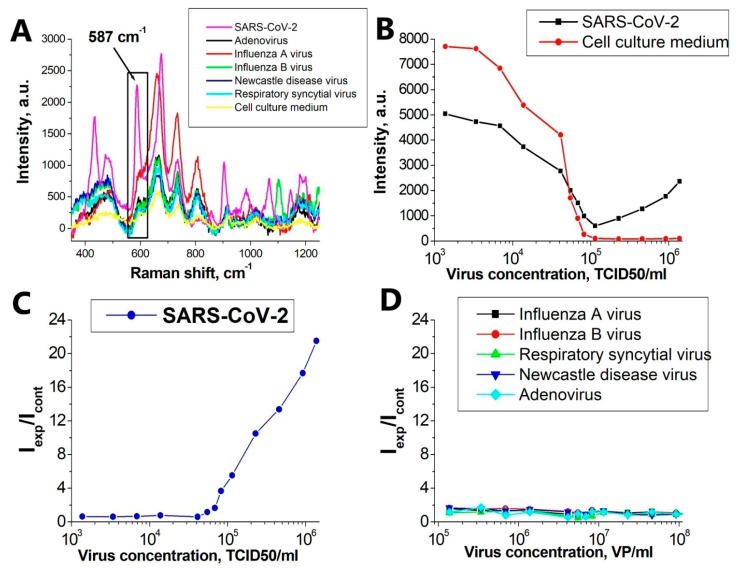
Detection of SARS-CoV-2. (**A**) SERS spectra of an experimental virus sample (SARS-CoV-2) at a concentration of 4.6 × 10^5^ TCID50/mL; virus-free cell culture medium in the same dilutions and control viruses in the same concentrations. (**B**) The dependence of the peak intensity of 587 cm^−1^ on the concentration of SARS-CoV-2 and for the virus-free cell culture medium in the same dilutions. (**C**) Concentration curve for SARS-CoV-2. (**D**) Concentration curve for control viruses.

For each virus studied, the concentration dependence of the SERS signal was investigated. The cell culture medium was used as a control. In the observed SERS spectra in the experimental and control samples, the intensity of the main Raman peak of the Bodipy FL label 587 cm^−1^ was studied (Figure 4A). As a result, the concentration dependence of the intensity ratio of the SERS line (587 cm^−1^) in the experimental and control samples was constructed (Figure 4C,D).

Figure 4C demonstrates the performance of the system for detecting the inactivated SARS-CoV-2. The specificity was tested for RSV, IvA, IvB, NDV, and AdV. For qualitative comparison, control viruses were reduced to uniform concentrations and concentration curves were constructed for them. Calibration curve for SARS-CoV-2 is ranging from 5.5 × 10^4^ to 1.4 × 10^6^ TCID50/mL.

Our hypothesis explaining the behavior of the curves for SARS-CoV-2 and for non-specific viruses (Figure 4C) and describing the mechanism of SERS signal generation is as follows:

❖ It is known [31,32] that at high ionic strength, silver nanoparticles interact well with the surface proteins of the virus, forming aggregates on the virus particles. With an increase in the concentration of proteins, the aggregates of silver nanoparticles create inhomogeneities of the electromagnetic field with a locally high density («hot spots») near their surface.
❖ Due to significant differences in the number of the reporter molecules near non-specific viral particles and SARS-CoV-2, the SERS signal decreases with increasing concentration of the non-specific virus, and in the case of SARS-CoV-2, it increases.

**Figure 5:**
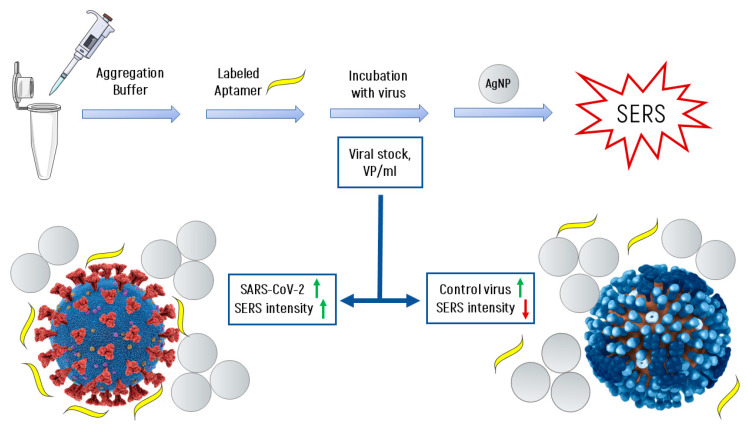
The scheme of the experiment and the formation of the SERS signal.

Figure 5 shows the scheme of the experiment and explains the principle of operation of the SERS-aptasensor. The target virus (SARS-CoV-2 virion, left side) accumulates more labeled aptamers due to the specific interaction mentioned earlier, while control viruses (i.e., influenza A virion, right side) have fewer labeled aptamers for the same enhancement field from AgNPs aggregates. This resultsin an increase of SERS intensity with higher concentrations of SARS-CoV-2 and in a decrease for control viruses.

## 4. Discussion

SARS-CoV-2 virus has many common symptoms inherent in other respiratory viral infections, therefore, it is often identified only in the late stages of the disease. In this regard, the creation of a simple and cheap method and device for its rapid detection may slow down such a rapid increase in the spread of the pandemic.

There are many different types of SERS substrates [33,34,35,36] and schemes for creating aptasensors based on them [20,37,38,39,40]. However, to create a quantitative method for the selective detection of SARS-CoV-2 from a group of other respiratory viral infections, this method should be the cheapest, simplest, and most reproducible. The reproducibility of SERS sensors can be achieved only in the case of using either nanoperiodic SERS structures obtained using electron lithography methods [41,42] (however, in this case, the cost of their production is high and therefore the method cannot compete with ELISA), or colloidal SERS solutions [23,43]. Colloidal solutions are easy to synthesize, homogeneous, and allow to work on a handheld Raman spectrometer with high laser radiation powers for a long exposure time.

In this paper, we have developed a very simple rapid method for determining SARS-CoV-2, based on the scheme of a direct method for assembling SERS aptasensor using colloidal SERS. The method presented in this article is simple («one-step»), fast (7 min), and has high sensitivity (LOD of 5.5 × 10^4^ TCID50/mL) and specificity.

Test specificity is a vital problem in diagnostics and the following treatment. In all existing detection methods, there is a trade-off between sensitivity and specificity. In the ideal situation, both diagnostic sensitivity and diagnostic specificity are 100%. However, the fact is that this situation is rarely reached and intention to increase the diagnostic sensitivity results in a decrease in diagnostic specificity. For tests in which false positives are unacceptable but false negatives may occur, high diagnostic specificity is required. It should be noticed that the more steps, materials, and hard-managed equipment a new method requires, the greater the degree of cross-reactivity in the approach.

Therefore, it is extremely important to develop new methods for selective and rapid detection of other viral respiratory diseases, such as: influenza virus, adenovirus, respiratory syncytial virus, and Newcastle disease virus. The authors of this article are already working on the creation of optical aptasensors for the selective detection of these viruses.

## Figures and Tables

**Table 1:** The parameters of the complexes of RBD of S-protein with aptamer RBD-1C and its shortened version, RBD-1C-sh. Kinetic constants of association (k_a_) and dissociation (k_d_) as well as equilibrium dissociation constants (K_d_) are provided.

Aptamer	k_a_, M^−1^s^−1^	k_d_, s^−1^	K_d_, M
RBD-1C	(1.7 ± 0.8)·10^5^	(2.17 ± 0.02)·10^−3^	(1.3 ± 0.2)·10^−8^
RBD-1C-sh	(1.7 ± 0.2)·10^4^	(5.6 ± 0.4)·10^−3^	(3.3 ± 0.6)·10^−7^

## Supplementary Materials

The following are available online at https://www.mdpi.com/article/10.3390/nano11061394/s1, Figure S1: UV-Vis spectroscopy of colloidal silver solutions, Figure S2: Dynamic Light Scattering (DLS) of colloidal silver solutions, Figure S3: SEM of AgNPs, Figure S4: ζ-potential of AgNPs, Figure S5: Spectra of the test substance (2 mkM xanthine) at different ionic strengths of the solution, Figure S6: UV-spectra of RBD-1C (during melting in potassium buffer).
