# Supplementary material for: SERS-Based Aptasensor for Rapid Quantitative Detection of SARS-CoV-2
Source: Nanomaterials (Basel). 2021 May 25;11(6):1394. doi: 10.3390/nano11061394 (PMC8228355; doi:10.3390/nano11061394)
Supplement: Supplementary file 1 [file nanomaterials-11-01394-s001.zip › nanomaterials-1224785-supplementary.pdf]

SERS-Based Aptasensor for Rapid Quantitative Detection of SARS-CoV-2

Elena Zavyalova[1,*], OganesAmbartsumyan[2],Gleb Zhdanov[1], Dmitry Gribanyov[3], Vladimir Gushchin[4], Artem Tkachuk[4], Elena Rudakova[5], Maria Nikiforova[4], Nadezhda Kuznetsova[4], Liubov Popova[4], Bakhtiyar Verdiev[4], Artem Alatyrev[4], Elena Burtseva[4], Anna Ignatieva[4], Anna Iliukhina[4], Inna Dolzhikova[4],
Alexander Arutyunyan[6], Alexandra Gambaryan[7] and Vladimir Kukushkin[3,*]

1 Chemistry Department, Lomonosov Moscow State University, 119991 Moscow, Russia; gleb.zhdanov@chemistry.msu.ru
2 Department of Microbiology, Virology and Immunology, I.M. Sechenov First Moscow State Medical
University, 125009 Moscow, Russia; ogan-mail@mail.ru
3 Institute of Solid State Physics of Russian Academy of Science, 142432 Chernogolovka, Russia;
digrib@gmail.com
4 National Research Center for Epidemiology and Microbiology named after the honorary academician N. F. Gamaleya, 123098 Moscow, Russia; wowaniada@gmail.com (V.G.); artem.p.tkachuk@gmail.com (A.T.); marianikiforova@inbox.ru (M.N.); nadyakuznetsova0@gmail.com (N.K.); ljubovprokudina@gmail.com (L.P.); yuryevpolskei@yandex.ru (B.V.); artem.alatyrev@gmail.com (A.A.); elena-burtseva@yandex.ru (E.B.); valgella@yandex.ru (A.I.); sovanya97@yandex.ru (A.I); i.dolzhikova@gmail.com (I.D.)
5 Institute of Physiologically Active Compounds of Russian Academy of Science, 142432 Chernogolovka, Russia; evladru@mail.ru
6 Belozersky Research Institute of Physico-Chemical Biology, Lomonosov Moscow State University,
119991 Moscow, Russia; Alarut@genebee.msu.ru
7 Chumakov Federal Scientific Center for Research and Development of Immune and Biological Products RAS, 108819 Moscow, Russia; al.gambaryan@gmail.com
* Correspondence: Correspondence: zlenka2006@gmail.com (E.Z.); kukushvi@mail.ru (V.K.)

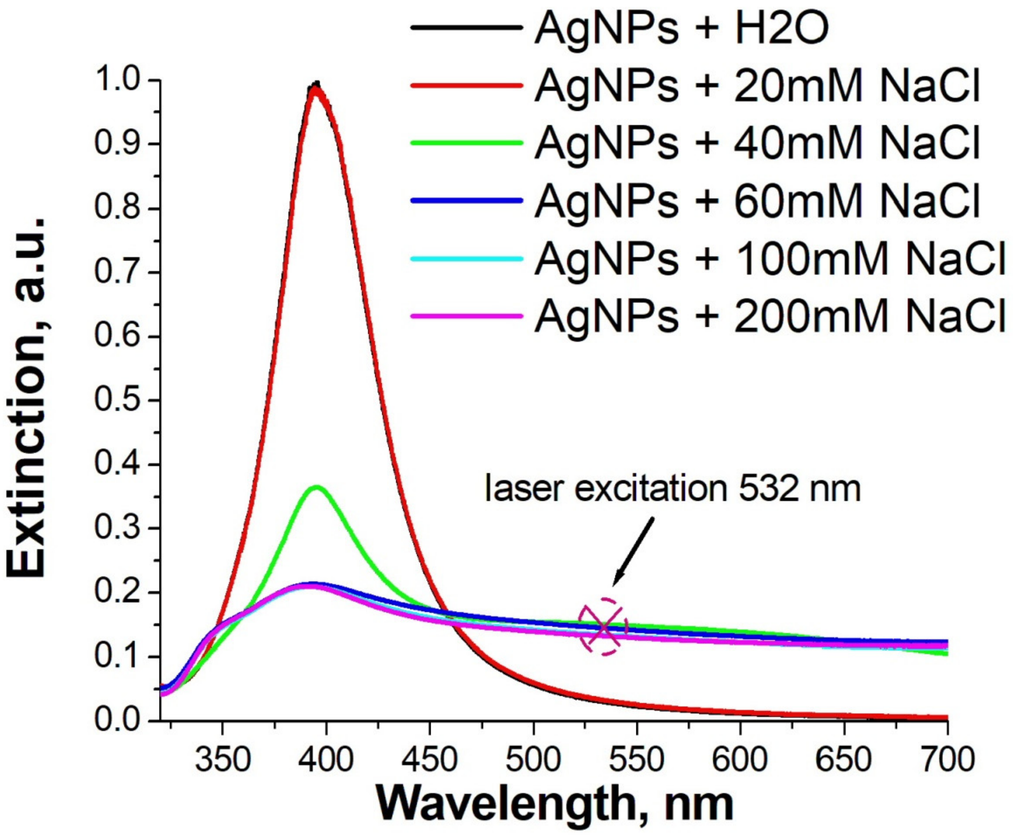

Figure S1. UV-Vis spectroscopy of colloidal silver solutions.

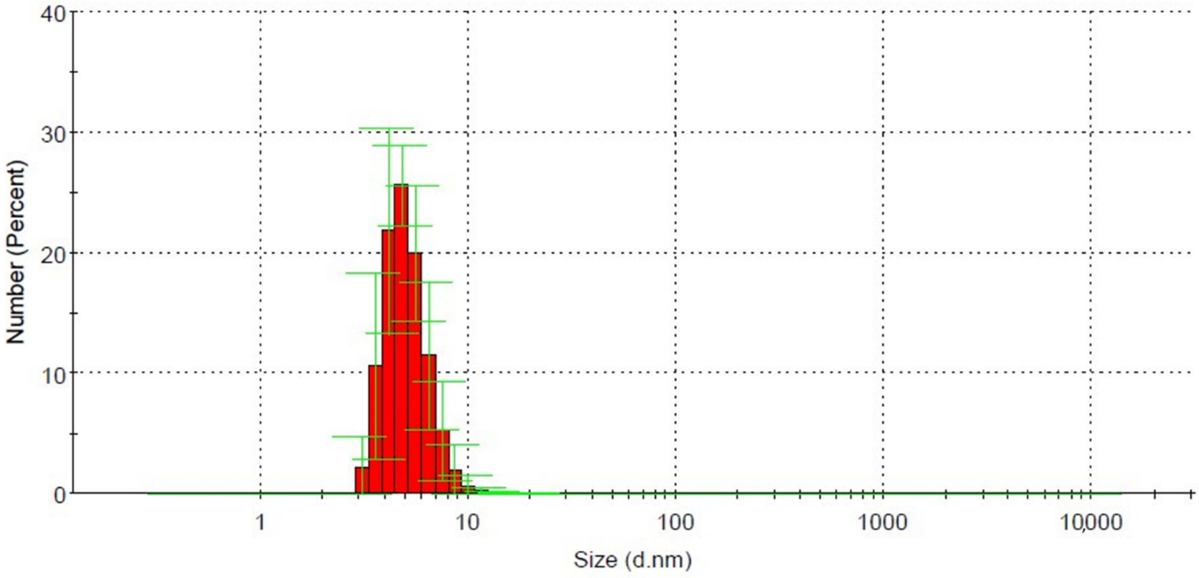

Figure S2. Dynamic Light Scattering (DLS) of colloidal silver solutions.

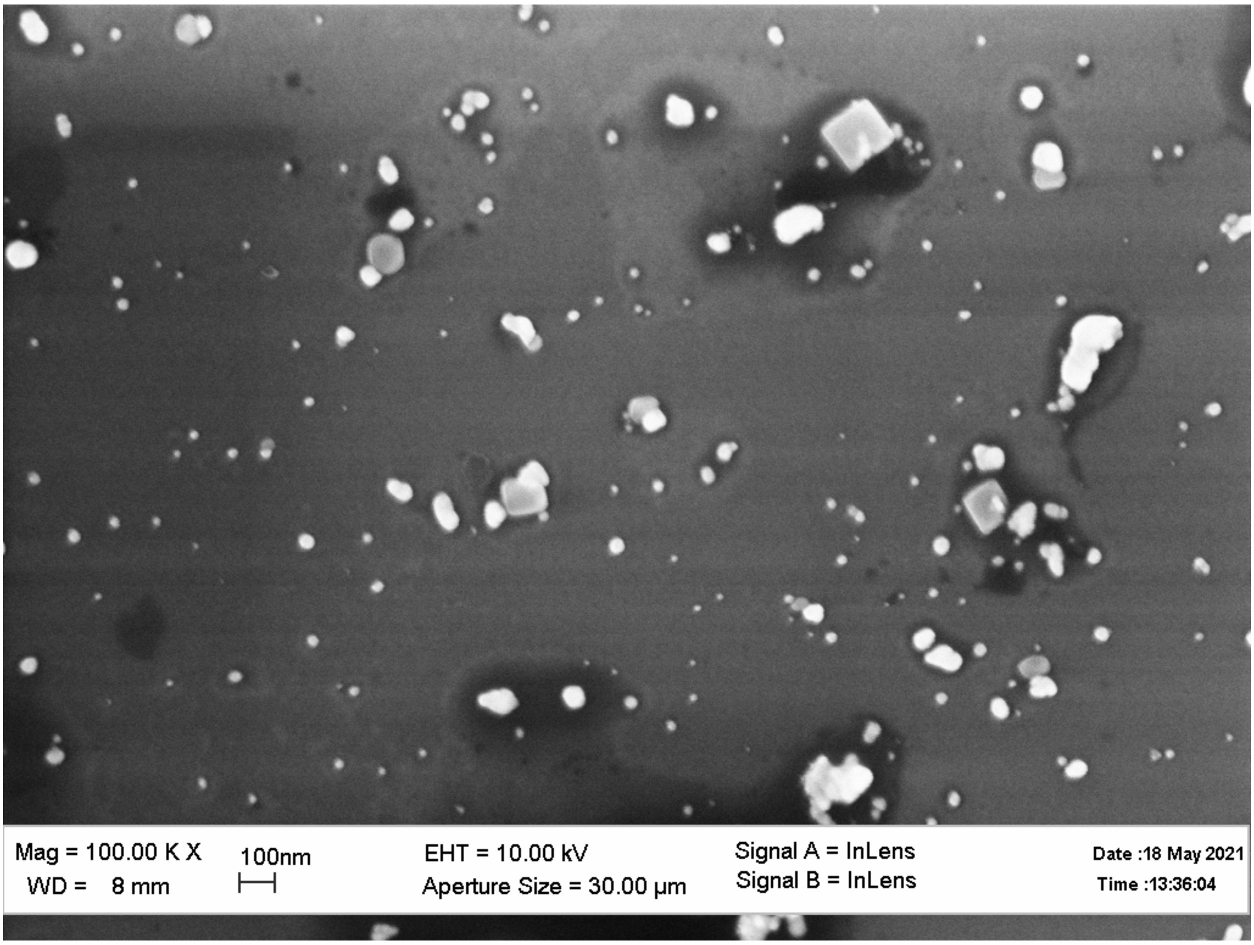

Figure S3. SEM of AgNPs

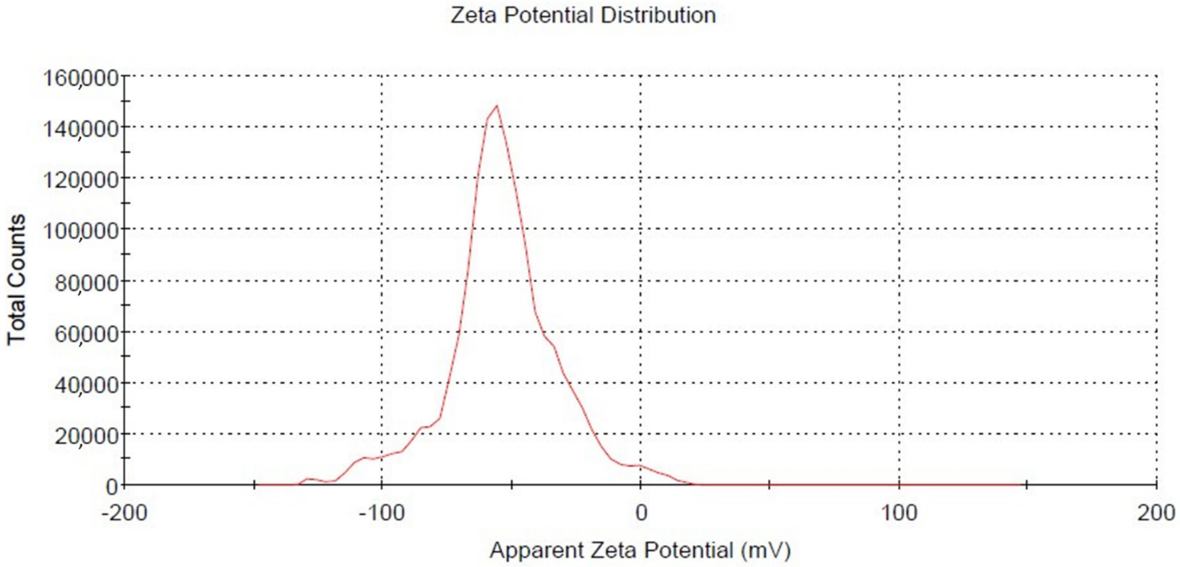

Figure S4. ζ-potential of AgNPs

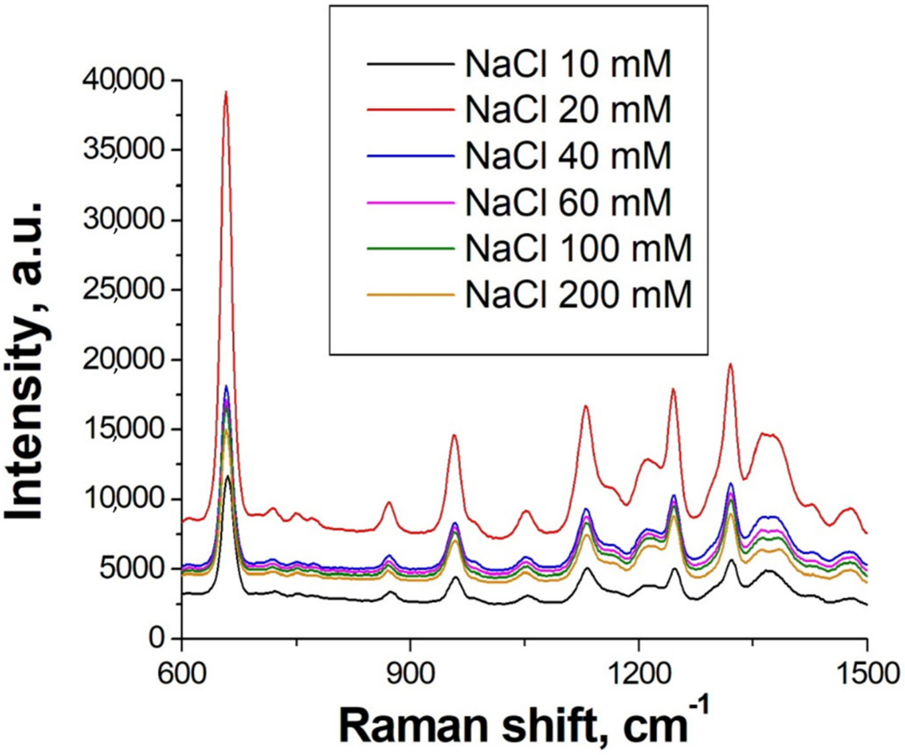

Figure S5. Spectra of the test substance (2 mkM xanthine) at different ionic strengths of the solution.

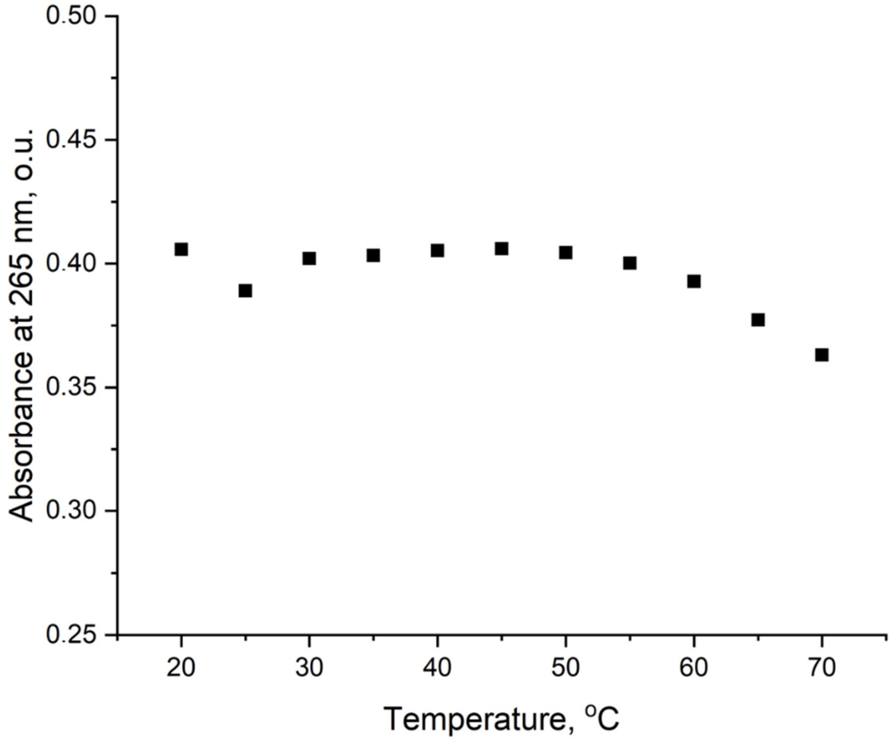

Figure S6. UV-spectra of RBD-1C (during melting in potassium buffer).
